# Aerobic endurance training to improve cognition and enhance recovery in schizophrenia: design and methodology of a multicenter randomized controlled trial

**DOI:** 10.1007/s00406-020-01175-2

**Published:** 2020-08-03

**Authors:** Isabel Maurus, Alkomiet Hasan, Andrea Schmitt, Astrid Roeh, Daniel Keeser, Berend Malchow, Thomas Schneider-Axmann, Martin Hellmich, Sabine Schmied, Moritz Lembeck, Katriona Keller-Varady, Irina Papazova, Dusan Hirjak, Cristina E. Topor, Henrik Walter, Sebastian Mohnke, Bob O. Vogel, Wolfgang Wölwer, Frank Schneider, Karsten Henkel, Andreas Meyer-Lindenberg, Peter Falkai

**Affiliations:** 1 Department of Psychiatry and Psychotherapy, University Hospital, LMU Munich, Nussbaumstrasse 7, 80336 Munich, Germany; 2 Department of Psychiatry and Psychosomatics of the University Augsburg, Bezirkskrankenhaus Augsburg, University of Augsburg, Augsburg, Germany; 3 Laboratory of Neuroscience (LIM27), Institute of Psychiatry, University of Sao Paulo, São Paulo, Brazil; 4 Department of Radiology, University Hospital, LMU Munich, Munich, Germany; 5 Department of Psychiatry and Psychotherapy, University Hospital Göttingen, Göttingen, Germany; 6 Faculty of Medicine, Institute of Medical Statistics and Computational Biology, University Hospital Cologne, University of Cologne, Cologne, Germany; 7 Faculty of Medicine, Clinical Trials Centre Cologne, University Hospital Cologne, University of Cologne, Cologne, Germany; 8 Institute of Sports Medicine, Hannover Medical School, Hannover, Germany; 9 Medical Faculty Mannheim, Central Institute of Mental Health, Heidelberg University, Heidelberg, Germany; 10 Department of Psychiatry and Psychotherapy, Charité Universitätsmedizin Berlin, Corporate Member of Freie Universität Berlin, Humboldt-Universität Zu Berlin, and Berlin Institute of Health, Berlin, Germany; 11 Department of Psychiatry and Psychotherapy, Medical Faculty, Heinrich-Heine University, Duesseldorf, Germany; 12 University Hospital, Heinrich-Heine University, Düsseldorf, Germany; 13 Department of Psychiatry, Psychotherapy and Psychosomatics, Faculty of Medicine, RWTH, Aachen, Germany

**Keywords:** Randomized clinical trial, Schizophrenia, Exercise, Physical activity, Cognition, Recovery

## Abstract

Even today, patients with schizophrenia often have an unfavorable outcome. Negative symptoms and cognitive deficits are common features in many patients and prevent recovery. In recent years, aerobic endurance training has emerged as a therapeutic approach with positive effects on several domains of patients’ health. However, appropriately sized, multicenter randomized controlled trials that would allow better generalization of results are lacking. The exercise study presented here is a multicenter, rater-blind, two-armed, parallel-group randomized clinical trial in patients with clinically stable schizophrenia being conducted at five German tertiary hospitals. The intervention group performs aerobic endurance training on bicycle ergometers three times per week for 40–50 min/session (depending on the intervention week) for a total of 26 weeks, and the control group performs balance and tone training for the same amount of time. Participants are subsequently followed up for 26 weeks. The primary endpoint is *all-cause discontinuation*; secondary endpoints include psychopathology, cognition, daily functioning, cardiovascular risk factors, and explorative biological measures regarding the underlying mechanisms of exercise. A total of 180 patients will be randomized. With currently 162 randomized participants, our study is the largest trial to date to investigate endurance training in patients with schizophrenia. We hypothesize that aerobic endurance training has beneficial effects on patients’ mental and physical health, leading to lower treatment discontinuation rates and improving disease outcomes. The study results will provide a basis for recommending exercise interventions as an add-on therapy in patients with schizophrenia.The study is registered in the International Clinical Trials Database (ClinicalTrials.gov identifier [NCT number]: NCT03466112) and in the German Clinical Trials Register (DRKS-ID: DRKS00009804).

## Introduction

The typical onset of schizophrenia in late adolescence or early adulthood and its low remission rates and significant negative effects on everyday life represent a substantial burden for patients [1, 2]. In about 75% of patients, the disease course is characterized by a sequence of remitting phases of positive symptoms [3]. Antipsychotics have proven to be effective in treating positive symptoms. However, negative symptoms and cognitive deficits often persist, despite guideline-based treatment [4, 5]. Because negative symptoms and cognitive deficits play a crucial role in impaired occupational and social functioning [6, 7], they often prevent sustainable recovery. Recovery is a multidimensional construct that includes a range of clinical and functional outcomes (e.g., education, employment, and relationships). According to a meta-analysis, only one in seven individuals with schizophrenia meets the criteria for recovery and shows clinical and social improvements over a period of at least 2 years [3]. Despite intensive and ongoing research, the proportion of cases attaining a good outcome has not increased [8]. Therefore, novel treatment options are required that foster the different facets of recovery.

In recent years, exercise has emerged as such a possible new treatment option. Meta-analyses showed that exercise interventions are effective in improving negative and general symptoms, cognition, global functioning, and quality of life in patients with schizophrenia [9, 10]. However, the underlying studies were small, single center, and heterogeneous. Although many open questions remain about the neurobiological mechanisms responsible for the effects of aerobic endurance training in schizophrenia patients, researchers assume that this type of training enhances neuroplasticity at the morphological and functional levels by modulating the concentrations of neurotrophic factors and neurotransmitters [11].

Beyond improving patients’ mental state, exercise also enhances their physical well-being and health. Patients with schizophrenia consistently have higher morbidity and mortality than the general population [1, 12], in part due to an increased risk for somatic comorbidities such as cardiovascular diseases [13], metabolic syndrome [14], and diabetes [12, 15]. Unhealthy lifestyle habits contribute to the development of these conditions: Compared with the general population, patients with schizophrenia are less physically active [16], have unhealthy eating habits and smoke more [17, 18]. In sum, patients’ life expectancy is up to 20 years shorter than that of the general population [12, 19–21]. As a countermeasure, in its guideline “Management of Physical Health Conditions in Adults with Severe Mental Disorders”, the World Health Organization (WHO) recommends behavioral lifestyle interventions in all patients with severe mental disorders and cardiovascular risk factors or who are at risk of becoming overweight [22]. Moreover, the recently published Lancet Psychiatry Commission on physical health interventions for people with mental illness specifically emphasizes the importance of supervised exercise training sessions through mental healthcare [23]. Physical activity prevents cardiovascular diseases and correlates with a reduced mortality in this population [24, 25].

We previously conducted two proof-of-concept studies that were based on findings from animal studies demonstrating a neuroplastic effect of aerobic exercise in rodents [26, 27]. In our first study, we tested neurocognitive performance in eight male patients with schizophrenia and eight male healthy individuals before and after 12 weeks of endurance training consisting of three 30-min sessions per week on a bicycle ergometer. As a control intervention, eight male patients with multiepisode schizophrenia played table soccer for the same amount of time. The main finding was an increase in hippocampal volume after endurance training in both the patients with schizophrenia and the healthy individuals but no increase in hippocampal volume in the controls. Moreover, the patients with schizophrenia in the intervention group showed improvements in short-term memory and negative symptoms [26]. In a subsequent study with a similar design but additional cognitive training after 6 weeks and a larger sample of 21 patients with schizophrenia, we observed increases in psychosocial function and cognitive performance, as well as an increase in the volume of the left temporal gyri [27, 28]. In addition, we found volume changes in the left cornu ammonis 4/dentate gyrus and showed that these changes were influenced significantly by schizophrenia polygenic risk scores [29, 30].

A recent meta-analysis of 10 studies that investigated the effects of exercise on cognition in patients with schizophrenia showed positive results in global cognition (Hedges’ *g* = 0.33, *p* < 0.001) [31]. Another meta-analysis of 29 trials on the overall effects of exercise in patients with schizophrenia [9] found that exercise was superior to the control conditions in improving total symptom severity (*g* = 0.39, *p* < 0.001); positive (*g* = 0.32, *p* < 0.01), negative (*g* = 0.49, *p* < 0.001), and general symptoms (*g* = 0.27, *p* < 0.05); quality of life (*g* = 0.55, *p* < 0.001); global functioning (*g* = 0.32, *p* < 0.01); and depressive symptoms (*g* = 0.71, *p* < 0.001). However, study designs, diagnostic procedures, and outcome variables have varied widely in previous exercise studies in patients with schizophrenia [32, 33] and, so far, no multicenter randomized controlled trials have been conducted. The frequent lack of exact training parameters, a control group, a randomization procedure, or an adequately large sample size are the further shortcomings. Unfortunately, these issues have hampered the development of efficient exercise programs that have the greatest possible benefit in patients with schizophrenia and allow the underlying mechanisms of the effects of exercise to be investigated. Therefore, additional randomized controlled trials with large sample sizes and scientific training methodologies are urgently needed. The multicenter randomized controlled trial presented here was designed to meet that need using a proven, systematic study design, an extended duration, and an exercise training amount that is in accordance with current recommendations [34].

With this multicenter randomized controlled trial, we aim to validate the efficacy of endurance training by comparing the endpoint *all-cause discontinuation* in the intervention group and a control group performing balance and tone training. Secondary outcomes include cognitive performance, psychopathological symptoms, cost-effectiveness, and brain plasticity. We hypothesize that aerobic endurance training could contribute to a more favorable disease outcome and reduced impairment from the disease.

## Methods and design

The study presented here is a subproject of the research network ESPRIT (Enhancing Schizophrenia Prevention and Recovery through Innovative Treatments; coordinator: Andreas Meyer-Lindenberg), which is sponsored by the Federal Ministry of Education and Research (BMBF; funding identifier 01EE1407A). The aim of the research network is to develop diagnostic, therapeutic, and preventive concepts for schizophrenia on the basis of current knowledge about pathogenesis and its mechanisms, focusing on strategies with a solid preclinical evidence base that are not currently being pursued by commercial research efforts and are expected to be synergistic with guideline-conforming treatments to promote recovery.

Our study is being conducted at five sites in Germany. It is designed as a controlled, rater-blind, parallel-group randomized clinical trial with two arms. The intervention group performs aerobic endurance training on bicycle ergometers three times a week for 40–50 min (depending on the week of the intervention) for a total duration of 26 weeks; whereas, the control group performs balance and tone training, as described by Liu-Ambrose et al. [35], for the same amount of time. All raters and people performing the statistical analyses are blinded to the intervention group.

The study was approved by the local ethics committees (project nr. 706–15, date 18.05.2016) and registered at ClinicalTrials.gov (NCT03466112) and in the German Clinical Trials Register (DRKS00009804).

### Study sites

The following clinical trial sites are involved: Department of Psychiatry and Psychotherapy of the LMU Munich (coordinating site; Coordinating Investigator (CI): P. Falkai; Principal Investigator (PI): A. Hasan); Central Institute of Mental Health Mannheim (PI: A. Meyer-Lindenberg; Co-PI: D. Hirjak); Department of Psychiatry and Psychotherapy of the University Charité Berlin (PI: H. Walter); Department of Psychiatry and Psychotherapy of the University Düsseldorf (PI: W. Wölwer); and Department of Psychiatry and Psychotherapy of the University RWTH Aachen (PI: K. Henkel). All study personnel were trained for the intervention and outcome parameters.

Organizational project management is performed by the Studienzentrum Psychiatrie (SZP) of the LMU Munich. Monitoring, data management, and randomization are performed by the Clinical Trials Centre Cologne (CTC Cologne), an independent academic research organization.

### Study population

The study includes in- and outpatients (men and women) aged from 18 to 65 years with a primary diagnosis of schizophrenia according to the Diagnostic and Statistical Manual of Mental Disorders 4th Edition, Text Revision (DSM-IV-TR 295.10-30, 295.90). Participants are assessed by the Mini-International Neuropsychiatric Interview [36] (M.I.N.I. Version 6.0.0). Further inclusion criteria are the ability to provide informed consent; stable psychopathology, indicated by a total Positive and Negative Syndrome Scale (PANSS) score ≤ 75 (no minimum value); and treatment with one or two antipsychotics (including clozapine) in accordance with the current treatment guidelines. The active substance and dose of the antipsychotic medication with which the patient has reached psychopathological stability must be constant for at least 2 weeks prior to inclusion in the study. The additional administration of antipsychotics as rescue medication for restlessness and sleep disorders is permitted if the daily dosage does not exceed 150 chlorpromazine equivalents. Concomitant psychotherapy and other psychosocial treatments are allowed (if administered, details are noted). Additional inclusion criteria are a negative pregnancy test (serum) at baseline and use of a reliable method of contraception in female participants and a body mass index between 18 and 40 kg/m^2^.

Exclusion criteria are an inability to give informed consent; presence of suicidality or being a risk to others; severe somatic or neurological comorbidities; history or assumption of relevant non-compliance that interferes with the ability to participate in a clinical trial; positive urine drug screening (except benzodiazepines); lack of German language skills; and pregnancy or lactation.

With regard to the recruitment process, inpatients and outpatients are screened regularly at the study sites. In a next step, an investigator informs each potential participant (as well as the legal representative, if applicable) comprehensively in verbal and written form about the objectives, procedures and possible risks of the study. A signed informed consent is obtained from each participant (and legal representative, if applicable) prior to any study-related procedure.

### Interventions

#### Description and fidelity assessments

The exercise training design was based on the experiences and methodological principles of the previous exercise studies conducted by our research group [26, 27, 37]. Furthermore, to promote participants’ health in an optimal way, we adapted the training to the American College of Sports Medicine’s current recommendations on the length and intensity of interventions [34]. Patients with schizophrenia very often have only a low level of physical activity and are in a deconditioned state [14, 16, 38]. To minimize the risks associated with a sudden onset of physical training after previous inactivity, participants undergo a thorough examination before starting the training. A sports scientist supervises both the intervention and the control groups and is available at any time for guidance and support. All training sessions are led and accompanied by trained study staff. Moreover, the training intensity is increased gradually (as described below) to give the participants’ bodies sufficient time to adapt to the exercise. The frequency of three training sessions per week guarantees that participants have enough time to recover between sessions.

In general, training on bicycle ergometers is an easy form of movement that does not place too much stress on the joints and has no risk of falling [39]. In the control intervention, the risk of injury is kept as low as possible by avoiding the use of additional weights and selecting suitable exercises.

To increase the motivation of the participants, we address several aspects in both groups: One of the main incentives for people with severe mental illness to engage in exercise is improving physical health [40]. Therefore, we inform the participants that the training modalities have been adapted to that purpose. In addition, they are updated regularly about their progress regarding the training parameters. One of the most common barriers for engaging in exercise is the lack of social support [40]. However, supervised exercise in small training groups helps to establish a close relationship between participants and study staff, which contributes to maintain motivation and adherence. Overall, exercise supervision has been shown to result in significantly greater physical and psychological benefits [23]. Therefore, our intervention is conducted by qualified and trained personnel. Besides, the regular exercise appointments can be helpful to improve the participants' daily routine, which might also increase adherence.

After giving informed consent, participants complete the diagnostic, clinical, and neurocognition- and performance-related baseline assessments. Both groups undergo performance testing, which consists of a lactate threshold test on a bicycle ergometer. Participants start at 25 watts, then the wattage is increased by 25 watts every 3 min until the participant is exhausted or cardiovascular risk factors occur [according to 41]. The wattage and respective lactate values are recorded.

Both groups participate in three training sessions per week. To reduce the risk of injury and promote recovery, participants start and end each training session with a 5-min warm-up and 5-min cool-down phase at 80% of the intensity of the main training. The duration of the main part of the training sessions is increased after 6 and 12 weeks: The total duration of the sessions in weeks 1–6 is 40 min; in weeks 7–12, 45 min; and in weeks 13–26, 50 min. Trained study personnel accompany, control and document each training session in both groups by measuring heart rate and subjective exertion [according to 42] every 5–10 min and lactate concentration by random sampling every 4 weeks.

#### Aerobic endurance training (Intervention group)

The aerobic endurance training intervention consists of moderate-intensity general dynamic endurance training on bicycle ergometers performed in accordance with the continuous training method. The participants thereby maintain a certain wattage and remain in a predominantly aerobic metabolic state. Heart rate is measured continuously. The blood lactate concentration measured during the baseline lactate threshold test allows us to determine the wattage at which the participant has to cycle to achieve a lactate concentration of approximately 2 mmol/l, which signifies an aerobic metabolic state. The participant should find the training “slightly strenuous” but should be able to talk without being short of breath. The training intensity is maintained until week 12, when we retest the lactate threshold and adjust the intensity if necessary.

#### Balance and tone training (Control group)

Participants in the control group participate in balance and tone training, as described by Liu-Ambrose et al. [35]. This training combines stretching, mobility, stability, balance, and relaxation exercises, which are combined according to a standardized exercise catalogue. We chose this control method to minimize confounders (such as social interaction), which might influence the results. The intervention period, training duration, and training frequency are the same as in the experimental group. Similar to the experimental group, the participants perform a warm-up and cool-down phase and are supervised by a sports scientist, furthermore, the training documentation is based on the measured heart rate, lactate concentration and perception of performance. However, instead of regulating the training intensity according to the preceding results of the lactate threshold test, the main objective was to perform the exercises as correctly as possible. If a participant can perform the exercises over the entire required period of time with only little effort, the training personnel can suggest options to increase the difficulty of the exercises (e.g., crunching with arms folded behind the head instead of on the chest).

### Study timeline

**Fig. 1:**
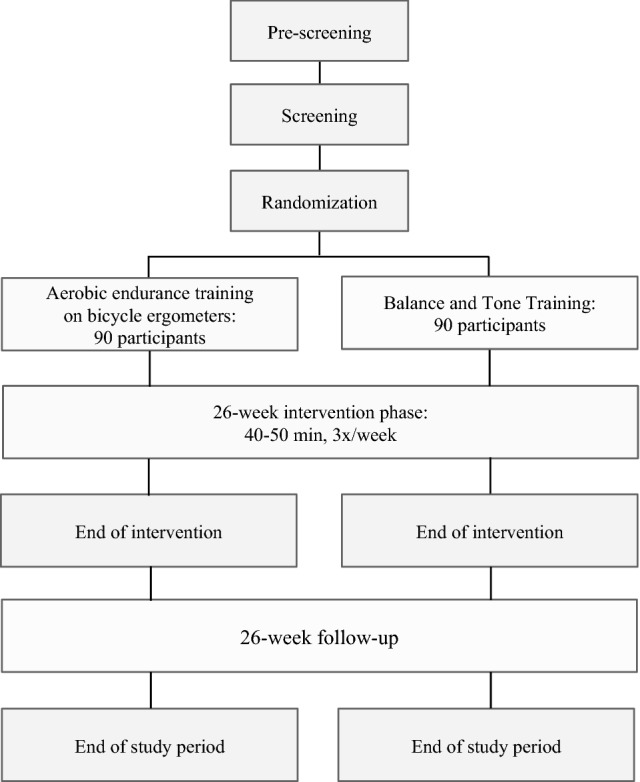
Flowchart of the study design I

**Fig. 2:**
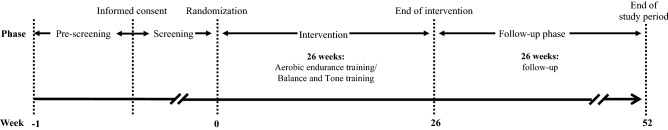
Flowchart of the study design II

The study flowchart is presented in Figs. 1 and 2. The pre-screening and screening phases can take place 1 week before randomization. The total study period after randomization includes a 26-week intervention period and a 26-week follow-up period (see Figs. 1 and 2). All the raters are blind to the participants’ intervention status. During the study and follow-up periods, a total of 10 visits are scheduled: The main examinations take place in the screening phase (visit 0), before the start of the intervention (visit 1), after 98 days of the intervention (visit 4), immediately after the end of the intervention (day 182, visit 6), and after the 26-week follow-up phase (day 365, visit 9); and the intermediate examinations take place on day 14 (visit 2), day 68 (visit 3), day 140 (visit 5), day 196 (visit 7), and day 273 (visit 8).

### Study endpoints

The primary endpoint is *all-cause discontinuation*, defined as relevant worsening of symptoms to a total PANSS score > 75 (on consecutive visits, for more than 14 days), severe adverse effects, non-compliance with the training program or diagnostic examinations for more than 6 weeks, an inability to reach the participant, or an unwillingness of the participant to continue participating in the study despite intensive efforts by the study team. The primary endpoint is evaluated at each visit. *All-cause discontinuation* is a comprehensive outcome that can be reliably assessed and reflects the participants’ satisfaction with the intervention and its tolerability and efficacy [43].

Secondary endpoints include changes in neurocognitive performance, psychopathology, level of functioning, quality of life, cardiovascular risk factors, metabolic parameters, and physical fitness. To measure neurocognitive performance, we assess verbal declarative memory with the German version of the Rey Auditory Verbal Learning Test (Verbaler Lern- und Merkfähigkeitstest, VLMT) [44],overall cognitive impairment and attention with the Digit Symbol Substitution Test (DSST) [45] and Digit Span Test [45],and verbal fluency with the Brief Cognitive Assessment Tool for Schizophrenia (B-CATS) [46]. Furthermore, we measure processing speed with the digit–symbol coding part of the DSST, and complex visual scanning, motor speed, and the ability to shift strategies with the Trail Making Tests A and B (TMT-A/B) [47]. Finally, we evaluate social cognition with the Facial Affect Recognition Test (PFA) and the Movie for the Assessment of Social Cognition (MASC) [48].

Psychopathological measures include the PANSS to assess positive and negative symptoms [49], Functional Remission of General Schizophrenia (FROGS) scale to assess functional remission status [50],Clinical Global Impression (CGI) scale to assess disease severity [51],and Calgary Depression Scale for Schizophrenia (CDSS) to assess depressive symptoms [52]. The level of general functioning is evaluated with the Global Assessment of Functioning scale (GAF) [53],Social and Occupational Functioning Assessment Scale (SOFAS) [54],Personal and Social Performance Scale (PSP) [55], and University of California San Diego Performance-based Skills Assessment (UPSA-B) [56]. Quality of Life is assessed with the World Health Organization Quality of Life-Bref (WHOQOL-Bref) [57], and compliance, with the Drug Attitude Inventory (DAI) [58] and the Service Engagement Scale (SES) [59]. We also determine care costs, cost-effectiveness of the study intervention, and costs of quality-adjusted life years (QALYs) [60]. Furthermore, we investigate participants’ dynamic variation in symptoms, affect, stress reactivity, social context, and sleep with ecological momentary assessments [61].

Cardiovascular risk factors and metabolic parameters include heart rate, blood pressure, body mass index, waist circumference, triglyceride level, total cholesterol, high-density lipoprotein cholesterol, low-density lipoprotein cholesterol, fasting blood glucose, and hemoglobin A1c.

The assessment of physical fitness includes an ergometer-based lactate threshold test to assess endurance capacity. Additionally, we evaluate the level of physical activity with the self-report International Physical Activity Questionnaire (IPAQ) [62] and, if participants agree, with the Activ8 accelerometer as an objective measure.

Adverse events and serious adverse events are documented in accordance with the established definitions and legal requirements.

Safety measures include a physical examination, electrocardiography, and repeated laboratory assessments and measurements of blood pressure and heart rate. Before participants are enrolled in the study, we exclude pregnancy and acute drug abuse. As described above, the exercise training was designed to be tolerable even for participants inexperienced in exercise. Consequently, participation is not associated with a health risk. However, a doctor is always on call should any physical health problems occur during the training sessions.

The study sites are experienced in clinical trials and have adequate study infrastructure, including a principal investigator (PI), deputy PI, sports scientists, and other study personnel, all trained in good clinical practice. Before the start of the study, the local ethics committees reviewed and approved the protocol and other required documents. They also reviewed any amendments to the protocol. Study centers are trained for each version of the protocol.

We also perform some explorative assessments, including structural and functional magnetic resonance imaging (MRI). We hypothesize that the MRIs will show an increased volume in the temporal lobe and hippocampus [26, 28] and an improved white matter connectivity between prefrontal and hippocampal areas in the aerobic endurance training group, as was found in the previous studies [63–66]. Moreover, we assess genome-wide gene expression and its influence on outcome parameters, epigenetics, and proteomics, including plasma levels of brain-derived neurotrophic factor. The biological measures are optional and performed only if participants do not fulfill the additional exclusion criteria, such as metallic implants in the case of MRI.

### Power calculation

On the basis of experiences in the German competence network [67] and the European First Episode Schizophrenia Trial (EUFEST) study [68], we assumed a one-year discontinuation rate of 68% in the control group.

To demonstrate superiority of 20 percentage points in the one-year discontinuation rate in the intervention group by two-sided testing with an error probability of *α* = 0.05 and a power of 1 − *β* = 0.8 in the chi-square test, we have to include about *n* = 90 participants in each study arm (software used: Power and Sample Size Calculation [PS] 3.0.43, freely available at https://biostat.mc.vanderbilt.edu/PowerSampleSize). It should be noted that the expected number of missing values for the endpoint *all-cause discontinuation* is (close to) zero. In addition, the expected power of the corresponding hypothesis test (e.g., log rank test, Cox regression) is slightly greater if the respective event time is taken into account.

### Planned data analyses

The primary analysis is according to the intention-to-treat (ITT) (full analysis set) and includes all randomized participants; only (clearly ineligible) subjects randomized in error will be excluded.

To compare the distribution of the primary endpoint *all-cause discontinuation* between the two treatment groups, we use stratified (by center) Cox regression with main effects treatment (intervention vs. control group), antipsychotic therapy, participants age, and sex (no interactions) at two-sided significance level 5%. Moreover, the influence of the covariate duration of illness is explored.

To confirm the stability of the model adaptation, we will pool the data of centers with low recruitment rates (< 8 randomized participants).

We will test the assumption of proportional hazard functions by adding time-dependent covariables. In addition, we will apply methods for competing risks (such as nonadherence, insufficient efficacy, and side effects of the medication).

We will analyze changes in neurocognitive performance, psychopathological symptoms, level of functioning, quality of life, cardiovascular risk factors, metabolic parameters, and physical fitness with linear mixed models for repeated measurements (MMRM; main effects: group, center, sex, participants age, (possibly) duration of illness, time, individual antipsychotic therapy, and the interaction group × time) and generalized estimation equations (GEE), both between groups and over the course of the study. Adjustment for multiple testing will be based on the closure test principle, for example the sequentially rejective Bonferroni–Holm method [69, 70]. Depending on the distributional characteristics, we may apply non-parametric methods.

We will perform a subgroup analysis by sex, center, and individual antipsychotic therapy and will investigate the possibly moderating influence of patient characteristics, especially duration of education or severity of disease (CGI). Adverse events will be summarized and compared between study arms according to the type, relationship to study intervention, severity, and intensity. The analyzing statistician will be blinded while transforming and analyzing the data.

In a primarily explorative approach, we will analyze the gene expression, epigenetics, proteomics, and MRI data by multivariate methods (e.g., statistical parametric mapping [SPM], projection to latent structure [PLS], machine learning approaches).

A sensitivity analysis will investigate the structure of the missing values (e.g., missing completely at random [MCAR], missing at random [MAR], or not missing at random [NMAR]) and explore the influence of different strategies for handling missing values (e.g., different imputation methods). In addition, we will investigate whether there was a clustering effect due to identical therapists or centers [71].

### Study progress and outlook

The ESPRIT subproject presented here, which is being conducted at several German tertiary care hospitals with a targeted number of 180 participants, is the largest trial to date investigating the efficacy of aerobic endurance training in patients with schizophrenia. Recruitment began in June 2016 and, at the time of submission of this manuscript, is still ongoing. So far, 162 participants have given informed consent and were randomized. Moreover, it is the first multicenter study on this topic with an external monitoring and organizational structure, which will further increase the conclusiveness of the results. Upon completion of the study, the large number of participants will allow us to generate general recommendations on exercise as a treatment option for patients with schizophrenia. To develop recommendations for high-quality treatment guidelines, multi-center randomized controlled trials or meta-analyses of those trials are needed [72]. Currently, there is a lack of multi-center randomized controlled trials regarding the efficacy of exercise interventions in schizophrenia patients and available meta-analyses are based on single-center trials. Such single-center trials are a source of a possible selection bias and do not allow generalization of findings. Therefore, our trial will fill this gap and allow guideline developers to evaluate the efficacy of this exercise intervention with a high-level of evidence. High-quality treatment guidelines in turn are a necessary step to demand from national healthcare providers to strengthen implementation, to provide financial compensation for the necessary staff and structural measures to promote a wider dissemination of exercise interventions in mental healthcare.

The primary endpoint *all-cause discontinuation* will provide insights into the participants’ satisfaction with the intervention, as well as its tolerability and effectiveness. The study includes a comprehensive investigation of the effects of aerobic endurance training, for example on psychopathology, cognition, and cardiovascular parameters. It will also consider quality of life and the costs of the intervention. Moreover, to identify the underlying mechanisms of aerobic endurance training and the relationship of training to recovery, we will evaluate neurobiological parameters, including structural and functional MRI data, epigenetics, and proteomics, in a subsample of participants. In addition, we aim to clarify how individual risk factors in patients with schizophrenia mediate or moderate the neurobiological effects of exercise on brain and cognition. Through these investigations, we expect to gain substantial understanding of how exercise can enhance recovery and how we can best use this effect.

One of the main strengths of our study is that, in contrast to most previous exercise studies, it follows general recommendations for endurance training, i.e., the duration of the intervention and follow-up periods are considerably longer. We assume that this will increase the positive effects of the training on the mental and physical health of patients with schizophrenia. The precise, well-proven training tests will be useful in revealing which exercise intensity is tolerable and beneficial, even for participants with little or no previous experience of exercise. With an intervention time of 150-min training per week for half a year, the study will enable us to offer recommendations for the amount of training that is required to show the desired effects. Furthermore, the 6-month follow-up will allow us to analyze the long-term effects of exercise interventions.

In conclusion, we hypothesize that upon completion, the study will support efficiently and effectively integrating exercise as an important element in the treatment concept for patients with schizophrenia to promote their recovery.

## References

[1] James SL (2018). Global, regional, and national incidence, prevalence, and years lived with disability for 354 diseases and injuries for 195 countries and territories, 1990–2017: a systematic analysis for the Global Burden of Disease Study 2017. The Lancet.

[2] Charlson FJ (2018). Global epidemiology and burden of schizophrenia: findings from the global burden of disease study 2016. Schizophr Bull.

[3] Jääskeläinen E (2012). A systematic review and meta-analysis of recovery in schizophrenia. Schizophr Bull.

[4] Nielsen RE (2015). Second-generation antipsychotic effect on cognition in patients with schizophrenia—a meta-analysis of randomized clinical trials. Acta Psychiatr Scand.

[5] Leucht S (2017). Sixty years of placebo-controlled antipsychotic drug trials in acute schizophrenia: systematic review, Bayesian meta-analysis, and meta-regression of efficacy predictors. Am J Psychiatry.

[6] Fusar-Poli P (2015). Treatments of negative symptoms in schizophrenia: Meta-analysis of 168 randomized placebo-controlled trials. Schizophr Bull.

[7] Villalta-Gil V (2006). Neurocognitive performance and negative symptoms: are they equal in explaining disability in schizophrenia outpatients?. Schizophr Res.

[8] Vita A, Barlati S (2018). Recovery from schizophrenia: Is it possible?. Curr Opin Psychiatry.

[9] Dauwan M (2016). Exercise improves clinical symptoms, quality of life, global functioning, and depression in schizophrenia: a systematic review and meta-analysis. Schizophr Bull.

[10] Dauwan M (2019). Physical exercise improves quality of life, depressive symptoms, and cognition across chronic brain disorders: a transdiagnostic systematic review and meta-analysis of randomized controlled trials. J Neurol.

[11] Maurus I (2019). Neurobiological effects of aerobic exercise, with a focus on patients with schizophrenia. Eur Arch Psychiatry Clin Neurosci.

[12] Laursen TM (2019). Cause-specific life years lost among persons diagnosed with schizophrenia: Is it getting better or worse?. Schizophr Res.

[13] Li M (2014). Schizophrenia and risk of stroke: a meta-analysis of cohort studies. Int J Cardiol.

[14] Vancampfort D (2015). Risk of metabolic syndrome and its components in people with schizophrenia and related psychotic disorders, bipolar disorder and major depressive disorder: a systematic review and meta-analysis. World Psychiatry.

[15] Vancampfort D (2016). Diabetes mellitus in people with schizophrenia, bipolar disorder and major depressive disorder: a systematic review and large scale meta-analysis. World Psychiatry.

[16] Stubbs B (2016). How much physical activity do people with schizophrenia engage in? A systematic review, comparative meta-analysis and meta-regression. Schizophr Res.

[17] Kouidrat Y (2018). Disordered eating behaviors as a potential obesogenic factor in schizophrenia. Psychiatry Res.

[18] Sagud M (2018). Smoking in schizophrenia: an updated review. Psychiatria Danubina.

[19] Vancampfort D (2018). Aerobic exercise in people with schizophrenia, in the exercise effect on mental health, neurobiological mechanisms.

[20] Laursen TM, Munk-Olsen T, Vestergaard M (2012). Life expectancy and cardiovascular mortality in persons with schizophrenia. Curr Opin Psychiatry.

[21] Hjorthoj C (2017). Years of potential life lost and life expectancy in schizophrenia: a systematic review and meta-analysis. Lancet Psychiatry.

[22] World Health Organization (2018). Management of physical health conditions in adults with severe mental disorders.

[23] Firth J (2019). The Lancet Psychiatry Commission: a blueprint for protecting physical health in people with mental illness. Lancet Psychiatry.

[24] Warburton DE, Nicol CW, Bredin SS (2006). Health benefits of physical activity: the evidence. Can Med Assoc J.

[25] Ward MC, White DT, Druss BG (2015). A meta-review of lifestyle interventions for cardiovascular risk factors in the general medical population: lessons for individuals with serious mental illness. J Clin Psychiatry.

[26] Pajonk FG (2010). Hippocampal plasticity in response to exercise in schizophrenia. Arch Gen Psychiatry.

[27] Malchow B (2015). Effects of endurance training combined with cognitive remediation on everyday functioning, symptoms, and cognition in multiepisode schizophrenia patients. Schizophr Bull.

[28] Malchow B (2016). Effects of endurance training on brain structures in chronic schizophrenia patients and healthy controls. Schizophr Res.

[29] Papiol S (2019). Polygenic burden associated to oligodendrocyte precursor cells and radial glia influences the hippocampal volume changes induced by aerobic exercise in schizophrenia patients. Transl Psychiatry.

[30] Papiol S (2017). Polygenic risk has an impact on the structural plasticity of hippocampal subfields during aerobic exercise combined with cognitive remediation in multi-episode schizophrenia. Transl Psychiatry.

[31] Firth J (2017). Aerobic exercise improves cognitive functioning in people with schizophrenia: a systematic review and meta-analysis. Schizophr Bull.

[32] Malchow B (2013). The effects of physical exercise in schizophrenia and affective disorders. Eur Arch Psychiatry Clin Neurosci.

[33] Schmitt A (2019). Aerobic exercise in mental disorders: from basic mechanisms to treatment recommendations. Eur Arch Psychiatry Clin Neurosci.

[34] Garber CE (2011). Quantity and quality of exercise for developing and maintaining cardiorespiratory, musculoskeletal, and neuromotor fitness in apparently healthy adults: guidance for prescribing exercise. Med Sci Sports Exerc.

[35] Liu-Ambrose T (2010). Resistance training and executive functions: a 12-month randomized controlled trial. Arch Intern Med.

[36] Sheehan DV (1998). The Mini-International Neuropsychiatric Interview (M.I.N.I.): The development and validation of a structured diagnostic psychiatric interview for DSM-IV and ICD-10. J Clin Psychiatry.

[37] Keller-Varady K (2016). Endurance training in patients with schizophrenia and healthy controls: differences and similarities. Eur Arch Psychiatry Clin Neurosci.

[38] Eskelinen S (2017). Multiple physical healthcare needs among outpatients with schizophrenia: findings from a health examination study. Nord J Psychiatry.

[39] Kutzner I (2012). Loading of the knee joint during ergometer cycling: telemetric in vivo data. J Orthop Sports Phys Ther.

[40] Firth J (2016). Motivating factors and barriers towards exercise in severe mental illness: a systematic review and meta-analysis. Psychol Med.

[41] Steinacker JM, Liu Y, Reißnecker S (2002). Abbruchkriterien bei der Ergometrie. Deutsche Zeitschrift für Sportmedizin.

[42] Borg GAV, Noble BJ (1974). Perceived exertion. Exerc Sport Sci Rev.

[43] Fleischhacker WW (2005). The European First Episode Schizophrenia Trial (EUFEST): rationale and design of the trial. Schizophr Res.

[44] Helmstaedter C, Wietzke J, Lutz MT (2009). Unique and shared validity of the "Wechsler logical memory test", the "California verbal learning test", and the "verbal learning and memory test" in patients with epilepsy. Epilepsy Res.

[45] Tewes U, Hawie R (1991) Hamburg-Wechsler Intelligenztest für Erwachsene. Revision 1991. Verlag Hans Huber

[46] Hurford IM (2009). A brief cognitive assessment tool for schizophrenia: construction of a tool for clinicians. Schizophr Bull.

[47] Reitan RM, Wolfson D (1985). The Halstead-Reitan neuropsychological test battery: theory and clinical interpretation Reitan Neuropsychol.

[48] Chuleva S, Clausen H-J, Körner J (2009). Anwendung des MASC, eines neuen Instrumentes zur Erfassung sozialkognitiver Kompetenzen bei Jugendlichen. Praxis der Kinderpsychologie und Kinderpsychiatrie.

[49] Kay SR, Fiszbein A, Opler LA (1987). The positive and negative syndrome scale (PANSS) for schizophrenia. Schizophr Bull.

[50] Llorca P-M (2009). The “Functional Remission of General Schizophrenia” (FROGS) scale: development and validation of a new questionnaire. Schizophr Res.

[51] Guy W, ECDEU Assessment Manual for Psychopharmacology—Revised (1976) Rockville, MD: US Department of Health, Education, and Welfare. Public Health Service, Alcohol, Drug Abuse, and Mental Health Administration, NIMH Psychopharmacology Research Branch, Division of Extramural Research Programs, 1976, pp 218–222

[52] Addington D, Addington J, Maticka-Tyndale E (1993). Assessing depression in schizophrenia: the Calgary Depression Scale. Br J Psychiatry Suppl.

[53] Endicott J (1976). The global assessment scale. A procedure for measuring overall severity of psychiatric disturbance. Arch Gen Psychiatry.

[54] Morosini PL (2000). Development, reliability and acceptability of a new version of the DSM-IV Social and Occupational Functioning Assessment Scale (SOFAS) to assess routine social funtioning. Acta Psychiatr Scand.

[55] Schaub D, Juckel G (2011). PSP-Skala–Deutsche version der personal and social performance scale. Der Nervenarzt.

[56] Patterson TL (2001). UCSD Performance-Based Skills Assessment: development of a new measure of everyday functioning for severely mentally ill adults. Schizophr Bull.

[57] Group, TW (1998). The World Health Organization quality of life assessment (WHOQOL): development and general psychometric properties. Soc Sci Med.

[58] Awad AG (1996). Assessment of the patient's subjective experience in acute neuroleptic treatment: implications for compliance and outcome. Int Clin Psychopharmacol.

[59] Tait L, Birchwood M, Trower P (2002). A new scale (SES) to measure engagement with community mental health services. J Ment Health.

[60] Voß E, Salize HJ (2016). Health care utilization and cost-effectiveness analyses in prevention studies in the mental health care field. Ment Health Prev.

[61] Vogel B, Mohnke S, Walter H (2018). Ecological momentary assessment. Nervenheilkunde.

[62] van Poppel MNM (2010). Physical activity questionnaires for adults. Sports Med.

[63] Herting MM (2014). White matter connectivity and aerobic fitness in male adolescents. Dev Cogn Neurosci.

[64] Burdette JH (2010). Using network science to evaluate exercise-associated brain changes in older adults. Front Aging Neurosci.

[65] Voss MW (2010). Plasticity of brain networks in a randomized intervention trial of exercise training in older adults. Front Aging Neurosci.

[66] Voss MW (2016). Fitness, but not physical activity, is related to functional integrity of brain networks associated with aging. Neuroimage.

[67] Gaebel W (2011). Relapse prevention in first-episode schizophrenia–maintenance vs intermittent drug treatment with prodrome-based early intervention: results of a randomized controlled trial within the German Research Network on Schizophrenia. J Clin Psychiatry.

[68] Kahn RS (2008). Effectiveness of antipsychotic drugs in first-episode schizophrenia and schizophreniform disorder: an open randomised clinical trial. Lancet.

[69] Holm S (1979). A simple sequentially rejective multiple test procedure. Scand J Stat.

[70] Marcus R, Eric P, Gabriel KR (1976). On closed testing procedures with special reference to ordered analysis of variance. Biometrika.

[71] Boutron I (2008). Extending the CONSORT statement to randomized trials of nonpharmacologic treatment: explanation and elaboration. Ann Intern Med.

[72] Hasan A (2019). WFSBP guidelines on how to grade treatment evidence for clinical guideline development. World J Biol Psychiatry.

