# Correction to: Aerobic endurance training to improve cognition and enhance recovery in schizophrenia: design and methodology of a multicenter randomized controlled trial

**DOI:** 10.1007/s00406-021-01289-1

**Published:** 2021-07-05

**Authors:** Isabel Maurus, Alkomiet Hasan, Andrea Schmitt, Astrid Roeh, Daniel Keeser, Berend Malchow, Thomas Schneider-Axmann, Martin Hellmich, Sabine Schmied, Moritz Lembeck, Katriona Keller-Varady, Irina Papazova, Dusan Hirjak, Cristina E. Topor, Henrik Walter, Sebastian Mohnke, Bob O. Vogel, Wolfgang Wölwer, Frank Schneider, Karsten Henkel, Andreas Meyer-Lindenberg, Peter Falkai

**Affiliations:** 1 Department of Psychiatry and Psychotherapy, University Hospital, LMU Munich, Nussbaumstrasse 7, 80336 Munich, Germany; 2 Department of Psychiatry and Psychosomatics of the University Augsburg, Bezirkskrankenhaus Augsburg, University of Augsburg, Augsburg, Germany; 3 Laboratory of Neuroscience (LIM27), Institute of Psychiatry, University of Sao Paulo, São Paulo, Brazil; 4 Department of Radiology, University Hospital, LMU Munich, Munich, Germany; 5 Department of Psychiatry and Psychotherapy, University Hospital Göttingen, Göttingen, Germany; 6 Faculty of Medicine, Institute of Medical Statistics and Computational Biology, University Hospital Cologne, University of Cologne, Cologne, Germany; 7 Faculty of Medicine, Clinical Trials Centre Cologne, University Hospital Cologne, University of Cologne, Cologne, Germany; 8 Institute of Sports Medicine, Hannover Medical School, Hannover, Germany; 9 Medical Faculty Mannheim, Central Institute of Mental Health, Heidelberg University, Heidelberg, Germany; 10 Department of Psychiatry and Psychotherapy, Charité Universitätsmedizin Berlin, Corporate Member of Freie Universität Berlin, Humboldt-Universität Zu Berlin, and Berlin Institute of Health, Berlin, Germany; 11 Department of Psychiatry and Psychotherapy, Medical Faculty, Heinrich-Heine University, Duesseldorf, Germany; 12 University Hospital, Heinrich-Heine University, Düsseldorf, Germany; 13 Department of Psychiatry, Psychotherapy and Psychosomatics, Faculty of Medicine, RWTH, Aachen, Germany

## Correction to: European Archives of Psychiatry and Clinical Neuroscience (2021) 271:315–324 10.1007/s00406-020-01175-2

The article “Aerobic endurance training to improve cognition and enhance recovery in schizophrenia: design and methodology of a multicenter randomized controlled trial” written by Isabel Maurus, Alkomiet Hasan, Andrea Schmitt, Astrid Roeh, Daniel Keeser, Berend Malchow, Thomas Schneider-Axmann, Martin Hellmich, Sabine Schmied, Moritz Lembeck, Katriona Keller-Varady, Irina Papazova, Dusan Hirjak, Cristina E. Topor, Henrik Walter, Sebastian Mohnke, Bob O. Vogel, Wolfgang Wölwer, Frank Schneider, Karsten Henkel, Andreas Meyer-Lindenberg and Peter Falkai was originally published electronically on the publisher’s internet portal on August 3, 2020 without open access. With the author(s)’ decision to opt for Open Choice the copyright of the article changed to © The Author(s) 2020 and the article is forthwith distributed under a Creative Commons Attribution 4.0 International License, which permits use, sharing, adaptation, distribution and reproduction in any medium or format, as long as you give appropriate credit to the original author(s) and the source, provide a link to the Creative Commons licence, and indicate if changes were made. The images or other third-party material in this article are included in the article’s Creative Commons licence, unless indicated otherwise in a credit line to the material. If material is not included in the article’s Creative Commons licence and your intended use is not permitted by statutory regulation or exceeds the permitted use, you will need to obtain permission directly from the copyright holder. To view a copy of this licence, visit http://creativecommons.org/licenses/by/4.0/. Open Access funding enabled and organized by Projekt DEAL.

The original article has been updated.

